# Introductory Lecture before the Classes of the Chicago Medical College, Sept. 23, 1884

**Published:** 1884-11

**Authors:** J. E. Owens

**Affiliations:** Professor of Operative Surgery and Surgical Anatomy, Chicago Medical College, Medical Department Northwestern University, Chicago


### Article II.

### Introductory Lecture* before the Classes of the Chicago Medical College, Sept. 23, 1884. *By* J. E. Owens, M. D., *Professor of Operative Surgery and Surgical Anatomy, Chicago Medical College, Medical Department Northwestern University, Chicago.*

Gentlemen:—As the representative of the faculty of the Chicago Medical College, it becomes my pleasing duty, this evening, when we inaugurate the opening of another college year, to offer a cordial welcome to every student in this amphitheater,—those who are here for the first time; those who have advanced to the middle year, and those who have the gratification of counting the present session as the last of their collegiate curriculum. Since the close of last session several changes have been made in the faculty. The chair of physiology, left vacant by the resignation of Professor Gradle, has been filled by the appointment of Dr. R. W. Bishop. To Dr. Danforth, one of the professors of clinical medicine, has been assigned the duty of teaching you the pathology of renal diseases. Prof. Danforth has for many years not only made renal diseases a special study, but he has taught this branch in the more important medical colleges and hospitals in this city, and I congratulate you that you can avail yourselves of the opportunity of profiting by his teachings. Dr. W. E. Casselberry, whose appointment as lecturer last year was made too late to appear in the announcement for 1883–4, has, since the close of the last session, been elected by the trustees full professor of materia medica and therapeutics. Dr. W. W. Jaggard has been made adjunct professor of obstetrics. To Dr. Frank Billings' duties as demonstrator of anatomy, the faculty has added those pertaining to the office of lecturer on physical diagnosis. Dr. J. S. Marshall, whose lectures last winter were so acceptable, and who at that time had not received a formal appointment, has been elected lecturer on oral and dental diseases.

Dr. N. S. Davis, Jr., appears as lecturer on general pathology and pathological anatomy, working in your interests, under the direction of Prof. Christian Fenger, who has full charge of this department. Dr. Frank S. Johnson has received the appointment of lecturer on histology, acting under the direction of Dr. Lester Curtis, the professor of histology and practical microscopy.

These changes in the curriculum, together with the new appointments, have been made with a great deal of circumspection on the part of the faculty, with the view of adding to its efficiency and facilitating its work. You have confided to us a great trust, and it is doubtless a matter of extreme concern to you to know something of the principles which will guide us, and the methods that will aid us in preparing you for your future career.

The college which you have selected, gentlemen, has been in existence a quarter of a century, and this was celebrated last spring in a fitting manner.

The functions of a medical school are to train students to become medical practitioners, and to pass certain examinations which are the legal tests of their fitness to practice. The choice of the medical school should be a matter of great importance to you. Many elements are to be considered, the prestige of the school, the system of teaching, the moral influence, example and personal qualities of its teachers, the character and standing of its alumni. I can honestly assure you, gentlemen, that you, who have cast your lot with the Chicago Medical College, have made no mistake. You have entered a college that has the honor to have made the very first earnest, honest and successful effort for reform in medical teaching, in this country, by refusing to adopt the methods of education then and now in operation in almost all the medical colleges in the United States—methods which the profession and medical societies have for the most part vainly protested against for many years. By the defective method referred to, you, who find yourselves for the first time in a medical college, at the beginning of your studies, would be expected not only to listen to, but to understand the same lectures delivered to the most advanced students, namely, those who are applicants for the degree of Doctor of Medicine, in the last year of study. You can well imagine the difficulties you would encounter, and may also imagine how defective must be such medical training. In the Chicago Medical College, the system of instruction, as most of you know, comprises three consecutive annual courses of lectures. During the first year the student is instructed in anatomy, physiology, histology, practical microscopy and general chemistry, the very foundation of his medical education.

The information thus gained, in the first year, is applied in the second, and enables him to study intelligently, and to the very best advantage, the branches of study embraced in the second year. In a like manner, the members of the senior class are most thoroughly fitted to comprehend the subjects presented to them in the third year, which embrace the theory and practice of medicine, surgery, gynecology, diseases of children, ophthalmology, otology, nervous and mental diseases and medical jurisprudence.

In my opinion, the system enforced by this faculty is most admirable, and bear in mind, gentlemen, that to this college belongs the honor of priority in this matter in this country, and not only that, it is due this institution to announce that no other system than the one mentioned has ever been in force in this school. The student is left no choice of an easier but less efficient method. He is obliged to conform to these requirements of the Chicago Medical College or go to some other school. It has ever striven for the broadest and most liberal culture of its students, and to this end it has worked successfully for so long a period, never at any time having made any sacrifice of principles in order to obtain large classes. It has never held out such inducements as low fees, short terms, low order of attainments, but it has always worked to secure the best men. Its graduates leave the college as well educated for their life-work as those of any other college in the United States. And you, gentlemen, as so many have done before you, if you perform your part, shall be well prepared for holding places of honor in society and the profession.

This college has done *a great public service* by implanting amongst our people well-educated and worthy physicians and surgeons. It has done another good thing, namely, it has exerted a wholesome influence in *establishing* the *graded course* in *a few* of the medical schools of this country. Dr. Wm. A. Hammond, of New York city, a gentleman of world-wide reputation, in an article on medical education made use of the following language:

"Thus far but few medical colleges have done their full duty in the matter of arranging their courses of lectures so as to give the best possible advantages to the student.

"So far as I am aware," said he, "the only ones that have established graded courses are Harvard Medical School, in Boston, the University of Pennsylvania, in Philadelphia, the Chicago Medical College, in Chicago, the medical department of Syracuse University, in Syracuse, and the medical department of Yale College, in New Haven."

Gentlemen, I will suggest one change in this, namely, that inasmuch as the Chicago Medical College was the first to make this radical change in medical education, its name should stand at the head of this honorable list. The experience that you will gain here is partly derived from the system of instruction as between teacher and pupil. Some, in decrying lectures, which compose the greater part of the course, have advanced the idea that all can be acquired from books that is obtained in the class-room, and that the facts presented in lectures pass along too rapidly to produce a lasting impression. Such arguments are plausible, but unsound. Your teacher, in order to do the best by you, has gone over and analyzed a great mass of material for a single lecture. He has excluded all extraneous matter and controverted questions, epitomized as much as possible, and thrown in observations and discoveries not elsewhere to be found. It would require a vast extent of reading to cover anything like the ground occupied by a single lecture. The transitory impression depends upon inattention and want of skill in note-taking. The fault of most beginners is to attempt to note down too much. Endeavor to catch the words that are the key to the sentence, and write these down in the order in which they happen to fall. Take then the very first opportunity, while the subject is still fresh in your minds, to work them into a consistent whole. You thus apply your critical faculties to your notes, and the attention which this requires makes this knowledge your own. Let me advise you to study with a view to the acquisition of knowledge, and not merely to the passing of examinations. In addition to the daily class examinations or quizzes, this year the Faculty will still further advance the interests of the student by including recitations in the system of instruction. The daily quizzes are designed to gauge the amount of your knowledge, to correct its deficiencies, to make it thorough and accurate, and thus to ensure you safer progress in the study of medicine. By recitations we hope to *enforce* a closer individual application to the different branches, and to enable you the better to unravel the more difficult subjects. You must remember, gentlemen, that, when you have completed your studies in this institution, you will pass out into an immense professional field armed with the implements for your work. There you must compete with those who have gone before you. These are very numerous. I am told that in every community there are three or four doctors where one is really needed. Those succeed who are the best qualified. You can readily see, then, why this college gives so much thought to methods of instruction. If it is true that Canada has one physician for every 1,193 inhabitants, Austria one for every 2,500, Germany one for every 3,000, Great Britain one for every 1652, France one for every 1814, and the United States one, both regulars and irregulars, for every 600, with you it is a plain case of the "survival of the fittest."* Those who are to flourish must start well, and their success depends upon great skill, great tact and great industry. Let me remind you that you cannot begin too early to familiarize yourselves with the objects presented to you in the lecture room and at the clinics. At the hospitals you will, from the first, be required to see, listen, handle, smell, and, if need be, taste. The more you train the senses, the more delicate and discriminating they become.

The amount of information gained in this way cannot be overestimated, and it will continue to increase through life. In the hospitals, as well as in the college, you will become conversant with the various instruments of precision, the stethoscope, the ophthalmoscope, the laryngoscope, the sphygmo-

*The Physician Himself.

graph, the thermometer, the microscope and others. Here, gentlemen, you must learn to be gentle, circumspect and forbearing, for much of your work now and hereafter must be done in the midst of those who suffer, those with painfully exalted sensibilities, those whose minds and bodies are wearing from want of rest, and exhausted from disease. Let me urge you to observe every detail in the clinics. It is a common fault with students to pay no attention to the " little things." Too often, only the tissues as they seem to part at the touch of the knife, the flowing blood, the grand result, claim their attention. There is so much to learn before you come to the operation. Much may be learned by studying the expression of the patient, his movements, his manner, the condition of the internal organs, the bearing which organic diseases and functional disturbances have upon the results of operations. Early study to simplify complications. This is one of the essentials of success. If an operation is about to be performed, note the preparations, the position of the patient, the number of necessary assistants, their arrangement. Get a clear idea of the essential instruments employed in the individual operations, and also those—a kind of reserve corps — that *may* become necessary in possible emergencies. Do not consider the sponges, ligatures, basins, and other receptacles, beneath your notice. In this way you will learn to comprehend the certainties, the probabilities and the possibilities of every operation, and find it an easy matter to make such an arrangement and to take such precautions as will, when you become engaged in practice, bring you always full-handed and admirably prepared for your work. Disease is so expressive that a close observer, and one who is well trained, may make an unerring diagnosis at a glance ; in another instance, a few sounds fix exactly the condition of the diseased organ; in another, smell and taste become invaluable; in still others, touch and manipulation are aids that cannot be overestimated.

Indeed, I cannot conceive, after a student enters the lecture-room, or approaches the bed-side, that he can spare a moment from the special work before him. In the lecture-room, before the lecture begins, you may often find a few moments that can be well spent in reviewing, correcting, or rearranging the notes of the previous lecture. Remember that medicine is an honorable calling, and if you are not resolved to uphold that honor, you have mistaken your mission. Honor and duty require you to do right because it is *right*, and not for policy's sake. The dangers and temptations of student life in Chicago are many and varied. Next to those of high principles, that it is hoped all have instilled into them in earlier life, that earnest resolve to fill in this world the place of a man—the ever persevering application to the work before you, in mastering the many details and principles of the medical sciences, is the best safeguard. You are about to enter a society differing in many respects from any amongst which you have hitherto lived. From the very moment that you enter these halls you become in a certain sense members of the medical profession. You will be brought in daily contact with your professors, and other members of the fraternity. You will shortly become more or less intimately acquainted with each other. Similar aspirations and prospects will draw you together, and the friendships thus early formed will doubtless be amongst the sincerest and most lasting of your life.

It occurs to me that you must have an exceedingly inadequate conception of the profession of your choice—a profession whose members must cultivate science for its own sake, and must apply this science to the wants of the community, in encouraging the well being of mankind, by promoting the growth of a robust and healthy population, by searching out the causes of disease, and diminishing the rate of mortality. Why you have chosen this very liberal calling may not be clear to all of you. Certainly you have been influenced to take this step for a variety of reasons. Perhaps I can do you a service in detailing the salient points in the life of one most worthy of emulation. This is the more agreeable to me, inasmuch as the life of such a one is a scrap of the history of surgery.

Indeed, gentlemen, the very best wish I can entertain for you is that each one of you may become a Nélaton. Note his happy qualities.

* " Love of work, sustained by a firm will, a precocious maturity, a sure judgment, much " finesse," united to a rare good sense, and that gift of nature, desirable among all, and which one cannot acquire—a personal charm. Add to these, propitious circumstances, rendered more favorable still by his prudence and his moderation."

From the very beginning of his career, one could see him advancing with a steady step, and, without resting or stopping a moment, attain successively to every degree of success, appearing at last at the summit of French contemporaneous surgery. He acquired one of those reputations of which neither time nor distance renders us unmindful and which our fathers had never known.

Auguste Nélaton was born in Paris, June 17, 1807. He was the second son of Alexandre François Nélaton, upholsterer, and of Marie Louise Lauriau, daughter of a merchant of St. Denis. Soon after the birth of his son, the elder Nélaton entered the service of the army. In 1811, at the epoch of the

*J. Bédard.

Russian campaign, he left, at Saint Omer, where he was then residing, his wife and children, whom he was not to see again. At first the news from him came regularly, then more rarely, and finally ceased entirely. The resources of the family became finally exhausted. Then came days of struggle. But Mme. Nélaton was not one of those who remained inactive. She did needle-work, and, thanks to her energy and skill, the humble home was kept supplied. Happy the sons of those courageous women, whose good example engrafted in the heart of the child, upon the love of mother, a sentiment of duty and respect for work.

When Mme. Nélaton returned to Paris, in 1821, the times had improved. The son was placed in school. He gained there all those little distinctions so flattering to youth and indicative of success. It was there that he made the acquaintance of a tutor, who became finally a distinguished physician, M. Requin, then a student of medicine, and whose counsels probably decided the future of Nélaton.

In 1828, having finished his literary studies, he began the study of medicine. "Claude Bourdelin," says Fontenelle, in his Éloges, "was born in good circumstances, and hence he could live comfortably, although all the world enjoyed good health." A fine Greek scholar for his time, the physician of the Duchess of Burgundy was, however, more occupied in translating Pindar than in taking care of the sick. If there are a favored few who can live without work, there are others, less favored, who must work in order to live. Young Nélaton had a smattering of Greek, but, nevertheless, the recollection of his youth had left profitable lessons, and he appreciated the value of time.

Two years had hardly passed when he was made externe in the service of Dupuytren. Soon after, having come out successfully in the concors for interne, he had the good fortune to meet a master whose good sense was only equaled by his modesty, M. Baffos, who, convinced of the value of the experience which one acquires by hard work, extended to his pupils many clinical favors. Our new interne took care not to lose any opportunity that presented itself.

The asylum to which he was attached, being devoted to the care of sick children, offered a vast field for research. It was there that Nélaton gathered the materials for his inaugural thesis. Having collected his data from nature, his work on Tubercle of Bone, well deserved a place in surgical literature. For the first time that frightful evil was searched for in all parts of the osseous system. At that time Dupuytren, regarded for a long time as the greatest surgeon, possessed a prestige without an equal. The ambition of youth was to approach him, to become attached to him, one felt proud to be counted amongst his disciples, and this favor was much sought after. Temporarily absent from the Hotel Dieu, Nélaton did not neglect to follow the lessons of the master, and he longed for the moment when he could enter again the services of this great surgeon. He returned in the early part of 1835, but Dupuytren was no longer there. For some time sickness kept him away. He never reappeared, and died a month later, February 8, at the age of fifty-eight years. In the midst of the élite of the French schools, merit was counted at its just value. M. Nélaton was becoming known, he was attentively listened to, his knowledge valued, his approbation sought after. He had no haughty manners, the usual companion of presumptuous inexperience, but a modest demeanor based upon firmness and assurance.

"In the ward of the Hotel Dieu," said M. Diday, in charming pages, dictated by the recollection of early friendships, in the midst of future great men who were gradually getting a foothold in that legendary entre-sol, the entrance of Nélaton always created a sensation. One felt attracted towards this gentle young man by a peculiar charm mingled with respect." The old Hotel Dieu of which M. Diday speaks, with its dark wards, haunted by work and hope, no longer exists except in memory; but in the new sanctuary, to-day as formerly, a courageous and proud youth is always on the alert for an opening through which he may pass on to eminence. 1839 was a fortunate year. At the close of a double concours, he was named agrégé of the faculty of medicine and surgery of the School of Paris. He was then thirty-two years old. At that age success has an inexpressible charm, either because one feels it in a more lively manner, or because the imagination embellishes the present by the promise of a still more brilliant future. "The first lights of Aurora are not more dazzling than the first rays of glory."

The reputation of Nélaton was commencing to spread abroad, he could, however, still command his time. There was a period of about ten years, perhaps the most fruitful of his successful career, during which, thanks to an existence wisely regulated, he lived in continual association with science, devoted many hours to work, learned a great deal and prepared much for the future. At that time he conceived the idea of connecting his name with a great work, and undertook the publication of "Treatise on Surgical Pathology," of which he was only able to compose the first two volumes. In 1850 he entered the concours for the Chair of Operative Surgery, which gave M. Malgaigne to the faculty. The following year the decisive contest took place, at the termination of which he came into possession of the Chair of Clinical Surgery, upon which his teachings threw so much luster.

French surgery emerged from obscurity with Guy de Chauliac, about 1360. For nearly 200 years it was a profession forsaken by literary men, when there appeared the great Ambroise Paré. It was a long time after that, when there arose two men of first order, namely J. L. Petit and Desault. Between them they occupied a part of the eighteenth century, as Dupuytren filled the beginning of this. Coming after Dubois and Boyer, heir to the traditions of French art, brought up at the school of Hunter and Bichat, an experimenter at a time when physiology had hardly ceased to be speculative, admirably gifted for teaching, Dupuytren, in a few years, had become the first surgeon of his time. In the new impulse which his skill gave to surgery, the operative technique always held the first place. Equally sought after by the operator and the one operated upon, rapidity in execution became a necessity.

But this manual dexterity, greatly esteemed and very praiseworthy, especially at a time when the physician had not gained a complete mastery over pain, was in reality but a secondary quality. Surgery only became a veritable science the day that it emerged from the shop of the adroit barbers, and thus overstepped the limits of its etymology. It has long since reached a much higher plane. The operation itself is not the greatest of the services which surgery is able to render. The operative domain is but a bondage which science from day to day renders lighter and more endurable, seeking to free itself from a bloody tribute by becoming more and more the physician and less and less the surgeon. This should be the aim of every true surgeon.

Contemporary surgery crystallized this truth in a single word, Conservatism! Such is the motto of progress. Is it not a splendid eulogy of French surgery to say that it has contributed largely to this new evolution?

It is well known, Nélaton has said it himself, that the work on surgery which bears his name, and the publication of which he commenced, was continued and completed by his pupils.

In order to know him well, to judge him as he should be judged, we must follow him in another role. Early his taste carried him towards the practical side of science. Rare qualities had revealed themselves in his clinical conferences at St. Louis, and it was easy to foresee what he would one day become. When the doors of the faculty opened before him, he was forty-four years old. He was no longer a young man; still he was not of that age when, having nothing more to work for, the professor foresees repose in teaching. The end was not yet attained. He was still ambitious.

Let us advance with him. Nélaton was a thorough professor. Not that he had that wonderful faculty of expressing himself like most men of showy minds, who seduce rather than persuade, but it was his easy-flowing, unstudied language, slow and measured, that impressed his hearers. He knew so well how to say what was necessary, and he spoke to the point. Clearness, precision, exactitude, were his predominant qualities. "That which is not clear is not French," said Voltaire, and Nélaton was a true Frenchman.

Ordinarily those who have to be taught know nothing or very little, and the professor, in order to make himself understood, must first raise his audience to his level. He excelled in this. There is always a word better than any other that expresses a thought. The happy words came of themselves to his lucid mind, and, without seeking for rhetorical effect, he often succeeded in it. His clinical teaching was the constant object of his preoccupation. Frequently he was seen reappearing in the middle of the day, in the wards assigned him, in order to examine again, at his leisure, the patient that was to be the subject of the next day's lecture. Endowed with great knowledge, he connected the observations of the moment with analogous facts which he had witnessed, or of which he had read in the annals of science. Proceeding alternately by comparison and by induction, he at one time devoted himself to sounding the origin of the disease, at another time its symptomatic expression engaged his attention. He was thus enabled to get at its connections and affiliations, and soon became master of the problem, throwing light upon obscure points and removing all obstacles.

Such a lecture had for his audience the charm of improvisation, when it was really the fruit of long meditation and arduous research. From the beginning he had voluntarily adhered to this inflexible method. Such is the secret of his diagnostic powers which have so often astonished his audiences.

Let us note in his life that which he has stamped with progress. The very best work in reparative surgery is comparatively very imperfect. Inspired by the fine studies of one of his colleagues, he, in an operation for restoring a nose which lacked the osseous frame-work, included in his flaps the periosteum from the neighboring part, in order to give the newly made organ the desirable solidity. In closing an abnormal orifice, or reforming a canal where only a gutter remained, instead of borrowing a pediculated flap from the neighborhood exposed to the dangers of mortification, he cut a kind of bridge, attached on each side to the living parts by two bands, and the enclosing piece was put in place simply by sliding. His operations performed with a view to remedying the unfortunate contraction of a cicatrix, and his studies in secondary hemorrhage and many other subjects which we cannot now touch upon, attracted much attention. In the application of galvanic electricity to surgery, the thermic or galvanic cautery is most frequently employed. By the aid of a current of proper intensity enclosed in a handle, a piece of platinum in the shape of a knife is made to divide the tissues slowly and without hemorrhage. age. But the chemical action of the current can also be utilized. The needles, which represent the poles of the electrical apparatus, being inserted in the living parts, the interposed tissue closes the current. The passage of electricity determines in the parts traversed the phenomenon of electrolysis—that is to say, a separation of elements, a slow disorganization, or a kind of separating necrosis.

In 1864 Nélaton explained to the Society of Surgery the advantages of the then new method which may be called Italian. In a few sittings, he obtained a cure without pain or bloodshed of one of those deeply placed cervical tumors, which the knife could only reach at the price of dangerous mutilations.

He made the operation of opening the intestine for occlusion more certain in its results by first fixing the intestine to the lips of the abdominal wound, and opening it only after adhesion of parts had taken place.

In the operation for stone, he proposed, in order to avoid lesion of the bulb in endeavoring to secure a roomy opening, to carry the incision as far backwards as possible. He pointed out the danger of meddling with certain lymphatic tumors of the groin. He insisted upon the necessity of proceeding to the complete extirpation of cysts which develop beneath the hyoid bone, in order to guard againt their return. He showed that pulsatile tumors of bone are not aneurisms but simple varieties of osteo-sarcoma.

Instead of abandoning pelvic aneurisms, for which neither opening the sac nor compression of the vessel is possible, coagulating injections appeared to him indicated.

He called attention to a singular malady in which the foot ulcerates, wears out, so to speak, from without inward, on every part that touches the ground, including the bone. We are more dependent on the time in which we live than we think. We belong to it by education, by our ideas, by our emotions, by our friendships, by our recollections, and we could not be justly judged except through our own surroundings. Following that rugged path where incessant labor and painful trials are the price of success,—trained at the school of liberal teaching then flourishing,—in the midst of valorous men who, by example, cherished his hopes and sustained him in his work, he was one of those young men who, having emerged from the amphitheater a perfect anatomist, easily became a skillful surgeon.

A little later, Dupuytren shared the fruits of eminence with Nélaton, just as formerly Desault had shared them with Dupuytren. Both accomplished professors and clinicians, they made their reputations more by speech than by pen, and powerfully impressed the youth of their time with a seductive teaching. Endowed with a penetrating sight, with inventive minds, they showed themselves equally able in putting into practice all that could assist the supreme aim of their art, namely, Cure.

The one was gentle, open, accessible to all. The other was reserved, studied, impenetrable. There are men who seem born to rule others ; they recognize no master, want no equals, and suffer themselves to divide with no one.

Nélaton, more modest and generous, associated more than once with his young confreres in the merit of work. Having shared in the labor, he wished them to participate in the honor. He was well in advance of the olden times. Great changes which he had witnessed, and in which he had played a part, had taken place. To the surgery of yesterday, absorbed in perfecting operative procedures, had succeeded a surgery less impatient.

Our own country had just given the world a new wonder, which can only be compared to vaccination, namely, the momentary insensibility and happy forgetfulness which result from the inhalation of ether. Simpson, of Edinburgh, a few months later found in the vapors of chloroform a still more rapid but more dangerous anæsthetic. To suppress pain, to calm the fright which an operation excites, to avoid the painful anguish and shock which result from these influences, are some of the benefits of the, then, new discovery. The patients find in it other advantages, less apparent, perhaps, but really no less precious.

The surgeon, having no longer the spectacle of suffering before him, of which even the most hardened soul is not totally unmindful,—having now no longer the need of subduing the patient or controlling himself, he sets to work with a freer mind and a surer hand. No longer preoccupying himself with a celerity containing sometimes an element of danger, he can proceed with a prudent slowness, with more time to think of results, which should be, in fact, the predominant thought of the surgeon. Being the last resource, compelled by necessity, the operation is a new peril, to which we are only permitted to expose our patients on condition that we concentrate all our energies to secure a successful issue. An impalpable dust, suspended in the air, surrounds us. Rare at the tops of mountains and in the solitudes of the fields, organic molecules are disseminated in the air of cities and accumulate in the interior of our dwellings. The greatest accidents are to be feared amongst the wounded in crowded habitations, insufficiently ventilated. Protected on all sides by an epithelial cuirass, within as well as without, in the way of constant renewal, a healthy man offers to these invisible enemies a barrier for the most part inaccessible. A wound is an open frontier which must be defended, so much the more since the open door offers a double danger, namely, the ingress of the invisible enemy or the exit of our vital forces. From his début in hospital practice, Nélaton had been wrestling with those frightful complications of wounds whose mild forms are inflammation of the lymphatics, erysipelas, phlegmon, and whose highest expression is the deadly pyæmia. He had seen surgery, for a long time disarmed and submissive, finally enter into combat with this horrible plague.

Avoid as much as possible, said he, the open wound, and when it is necessary to have recourse to the knife, prevent in every way the introduction of the subtle poison by establishing quickly a protective barrier, and if, in spite of all, the septic agent appears at the surface of the wounds, try to expel it, or destroy it. The value of the means proposed is best seen when put in practice.

He was one of the first to use alcohol as a dressing for wounds, and personally interested himself in making known its value.

It is true, that, at a remote period when surgeons boasted of the most varied compositions, balms and mixtures of all kinds, to say nothing of the charms of the supernatural of Paracelsus, a few surgeons, bolder than others, treated wounds with hot water. But it is not progress to follow at random a path whose end we miss and which we lose as easily as we enter it. All the attempts of which we speak would be vain did not hygiene of the sick-room follow at the same time. Nélaton often insisted, in his teachings, upon the tried principles of prophylactic surgery. He taught that there are no "*small things*" in the art of curing, and that every detail has its importance, since the life of the patient may be endangered by a lotion badly made, or by an unclean element of the dressing.

To operate in good atmospheric conditions, to aim at rapid cicatrization—in a word, try to obtain immediate union, is the ideal of all reflective minds.

He took an active part in the surgical society, especially at the time of his début. He appeared but once or twice at the Academy. In 1856, on the occasion of his first appearance there, he took part in a hot discussion upon one of the gravest operations that can be performed upon woman, and of which the most independent, and, at the same time, the most incisive speaker, said, "That it would surely put the patient beyond the possibility of a relapse." This operation, that of ovariotomy, conflicted too violently with the existing ideas of that time, relative to the gravity of penetrating the peritoneal cavity, to be accepted without resistance. Ovariotomy was then looked upon as a kind of criminal procedure. When it came to France for the first time, from England, about 1840, it had already been practiced in our own country for more than thirty years. The first attempts made in France had not been fortunate. In England the most eminent surgeons condemned it. It was the repeated successes of Wells and Baker Brown that attracted anew attention to it.

November, 1861, wishing to see for himself, he went to England. Five operations made under his eyes by Baker Brown removed all his prejudices. He returned convinced. In his clinical lectures of the following year, he made an appeal in its behalf to his young colleagues. He, the prudent surgeon, par excellence, strove to dissipate exaggerated fears, and asked them to judge only after giving the operation a fair trial. Soon after, he detailed to the Academy the operation done by his esteemed confrère, M. Demarquay. It is not easily forgotten how much he insisted that they should not be discouraged by that first failure. Almost at the same time a young professor, Koeberlé, then his compatriot, successfully performed in Strasbourg a series of brilliant ovariotomies, which exerted a powerful influence in legitimizing the operation. It can be said that to Nélaton is due the honor of having acclimated this new American importation. If the operation, he thought, was to be dreaded, the disease was still more to be dreaded. Inevitable death is the fate of its victims. If the operation has its perils, it has also many chances of a happy result.

Said Nélaton, in one of his lectures, "I cannot too strongly urge you to lay aside sentimental prejudices." In the face of a disease, it is necessary, when the critical time has arrived, to put in parallel the gravity of the evil and the resources of our art. And, further on, he added this recommendation: "It is of great consequence that surgeons in high places should give their moral support to those of our colleagues who are willing to assume the responsibility of such operations."

The following year an unforseen event made the name of Nélaton renowned. August 29, 1862, at Aspromonte, Garibaldi, who was fighting for the independence and unity of his country, being in the front of the volunteer lines, was struck by three balls, at the first fire of the regular Italian troops. One contused the left knee, the other grazed the right hip, the third caused a more serious wound and penetrated the right ankle, a little above and in front of the internal mallēolus. Garibaldi took only a few steps, when, from pain, he was obliged to sit down, after which he made no further attempts to walk. A few minutes afterwards, on the battle field, Doctor Albanese, having noticed on the other side of the foot a slight swelling, made an incision at that point. Not having found the ball, he thought it prudent to wait. The wounded man was conveyed in a steamer to Spezzia, and entered the fort of Varignano on the evening of September 2d. Two days afterwards, in the presence of Rizzoli, Zannetti, Prandina, Negri, Riboli, and of Albanese, Repari and Bazile, physicians and friends of the general, Professor Porta, of Pavia, explored the wound. The consulting physicians concluded from this examination that the ball was not in the wound. M. Bazile, however, had some doubts on this point, and so expressed himself several times to Garibaldi, and also to Professor Porta. Nélaton was summoned. He arrived at Spezzia Tuesday, October 28th. The general had been confined to his bed two months. At the very commencement of the exploration he felt convinced that the ball was in the wound. The introduction of an ordinary probe gave to his equally trained ear and hand a double sensation, which left no doubt in his mind as to the presence of the ball. Such was also the opinion of Partridge and Pirogoff, who came three days afterward. Having returned to Paris, Nélaton reflected on the surest means of removing the doubts in the minds of his Italian colleagues, and he related with his accustomed modesty how he was directed in the right way. M. Emmanuel Rosseau, said he, gave him the hint. It consisted in introducing into the wound a body of small dimensions, capable of receiving a metallic impress easily recognized. A few days later, Nélaton sent to the surgeons of the general a probe having an olive-pointed, unpolished white porcelain extremity, upon which, by a rotary movement, the projectile revealed its presence. Professor Zannetti, who had failed with the electrical battery, obtained with this probe received from Paris the certainty so much desired. November 22d, a piece of compressed sponge was inserted in order to dilate the wound, and the next day Zannetti extracted the ball with ease.

The same day Nélaton received from Pisa the following telegraph, signed by the Prefect of Pisa, Torelli: "Ball extracted from Garibaldi's wound, verifying your diagnosis, guaranteed by your probe. All honor to you."

In the first part of December the general wrote to Nélaton: "*My very dear friend:* I owe you a few words of love and gratitude. Your coming to Spezzia has been a fortunate thing for me. If at any time a doubt had possessed my mind, your interview was so eminently sympathetic, your words of encouragement so eloquent, that I could no longer doubt my recovery. I am a great deal better since the extraction of the ball, so skillfully done by our illustrious compatriot, Professor Zannetti, by the aid of the instrument which you had the kindness to send me. May God bless you." "GARIBALDI."

The name of the wounded, the gravity of the event, of which Italy was the theatre, the eminence of those gentlemen who had held a different opinion, curiosity and public expectation, conspired to popularize the French surgeon. The name of Nélaton was spread far and wide, and he from this time took the first rank amongst his illustrious contemporaries. In our day, when talent abounds, individual renown is rare. At the time when he was called to Italy he enjoyed a reputation uncontested. After this event his success increased, and very soon his practice became immense. His prudence, his gentleness, the confidence which he inspired, the affectionate solicitude for his patients would have been alone sufficient without the merit of the surgeon, to explain his growing reputation. His frank and smiling face dissipated every fear. With him, rash undertakings were never feared. The confidence which he inspired was absolute. Full of kindness for his colleagues, he never intruded. There was in the medical corps no teacher more beloved or more sought after than he was. During the last ten years of his surgical practice there were but few difficult cases in which he was not called in consultation.

Toward the middle of 1867, M. Jobert being dead, Nélaton was called to the office of surgeon to the Emperor. To-day, when the idea of equality has become, as it were, a law of justice, the physician has no superior. Medicine demands a versatile talent, and a physician most worthy of the name represents the highest type of moral and intellectual culture. The social mission which he is called upon to fill, the extent and variety of knowledge which it exacts, the number and importance of its applications, assign him in our society a position which yields to none. His profession brings him in contact with every class of society, and it is his duty to proudly retain in his association with the powerful of the day what others lose, namely, sincerity and independence. In the office referred to, which he had not sought, Nélaton knew well how to foster in his own person a respect for the profession to which he belonged. On the very eve of the French disasters, the Emperor, in testimony of his gratitude, gave him a seat in that high chamber that was soon to disappear with imperial institutions. The preceding year he resigned his professorship. At the same time he tried to retire from that enervating work which he had endured so long. He was now 60 years old; his sight grew weak and his health began to fail. The surgeon acquires by strength of will the necessary *sang froid,* and a complete mastery of himself, without which he can do nothing. But in these noble efforts which do honor to the man, life oozes away from many wounds which are constantly renewed. However, events precipitated themselves. The day of the siege came. Nélaton desired to perform duty in the ambulance. This last effort broke down his health.

Cardiac disease, from which he was suffering, made rapid progress. His step became heavy, and an expression of languor and sadness had taken the place of the smile which formerly illuminated his face. Disturbances of the circulatory system were becoming more frequent. His visits became less and less. By unanimous vote, every year renewed, the physicians of the department of the Seine placed him at the head of their association. Although broken down by disease, he never ceased to coöperate in that brotherly work. Yielding to the solicitations of his relations and friends, he left Paris. But neither Italy, nor the sea, nor the air of the country could triumph over an incurable disease. On Sunday, September 21, 1873, he breathed his last, in the midst of his family, at the age of 66. His obsequies took place without pomp. No discourse was pronounced over his remains. This was his wish, and it was piously respected. Upon the hearse, which was surrounded by a sorrowing crowd, there was nothing to suggest the great dignitary of State, the academician or the professor. While they followed that modest coffin buried in flowers, the recollection of the eminent surgeon was involuntarily associated with one of the greatest French naturalists.

The surroundings were not those of luxury, but in a bare room, his hands chilled with misery, Michel Adanson requested, as the only decoration of his grave, a garland of flowers gathered from fifty-eight families of the vegetable kingdom he had differentiated—"a passing image," said Cuvier, "of the more durable monument which his own labors had erected for him."

Nélaton belonged to a hard working generation, for the most part passed away, worthy heir of the great surgeons oi the beginning of this century, a cultured élite which had rendered many services to the profession, not the least of which was the opening up of new paths still pursued by the surgeons of to-day. Experience is only acquired at the cost of many struggles. A stranger to all vulgar ambitions, not striving to eclipse any one, he had gained the esteem and affection of all.

He was already celebrated when his name became connected with the hero of Italian independence. In the succession of events which mark the course of life, the last are engraved most deeply in the memory. There are some whose written works stamp the names of their authors in the memory of generations. There are others, amongst the best, superior less by what they do than by what they are, more occupied in spreading truth by speech than by the pen, who seem to have worked more for their contemporaries than for posterity. From the past, what an amount of acquired knowledge has come into our possession, what varied discoveries—precious inheritance, whose origin is lost in the long ago, and which we spend without gratitude. Like those pieces of money whose figures have disappeared by long use, but which are still in circulation, without having lost their value. Do not forget that all real progress prepares the way for more. Science is an enchanting fugitive, of which it is said: "No one can seize it; it always allures us onwards, because it is always in advance of us."

